# Editorial: Tackling non-communicable diseases and epidemiological transition in low- and middle-income countries

**DOI:** 10.3389/fpubh.2026.1929613

**Published:** 2026-08-12

**Authors:** Annamari Doro Altan, Maria Rosaria Gualano, Fausto Ciccacci

**Affiliations:** 1 Department of Life Science, Health, and Health Professions, Link Campus University, Rome, Italy; 2 Unicamillus, Saint Camillus International University of Health Sciences, Rome, Italy; 3 Department of Biomedicine and Prevention, University of Rome Tor Vergata, Rome, Italy

**Keywords:** epidemiological transition, health system strengthening, integrated care, low-and lower-middle-income countries, non-communicable disease

The concept of epidemiological transition has long been used to describe changes in population health. In Omran's classic formulation, societies move from infectious diseases, famine, and high mortality toward profiles shaped by chronic non-communicable diseases (1). Subsequent reviews and contemporary analyses have refined this model, showing that epidemiological transitions across countries often follow heterogeneous and non-linear trajectories, particularly in LMICs (2, 3). Recent regional evidence from Africa has indicated that communicable and non-communicable diseases (NCDs) frequently coexist rather than replacing one another (4). NCDs are expanding while maternal and nutritional conditions, injuries, mental health needs, and social vulnerability continue to shape morbidity and mortality. The result is not replacement but rather coexistence, interaction, and accumulation. This double burden is visible at the population level and within individuals and households through disability, chronic morbidity, multimorbidity, delayed diagnosis, fragmented care, and social and environmental risks. Therefore, epidemiological transition in LMICs is increasingly a test of whether services can adapt to overlapping burdens, multimorbidity, and persistent inequities.

This Research Topic, “*Tackling non-communicable diseases and epidemiological transition in low- and middle-income countries*,” examines this challenge from multiple public health angles. The Research Topic brings together studies and perspectives on changing disease patterns, service delivery, community platforms, pharmacy-based screening, surveillance, registries, program indicators, implementation readiness, and policy frameworks. Its value lies not only in documenting the growing importance of NCDs, but in showing how services are being reorganized, and where this remains incomplete. Across diverse settings, the contributions herein ask how existing platforms can support prevention, early detection, continuity of care, referrals, monitoring, and accountability when resources are limited. Before considering responses, it is necessary to understand the uneven epidemiological landscapes to which these systems must respond.

Burden studies demonstrate that epidemiological transition cannot be treated as a uniform national trajectory. In Iraqi Kurdistan, for example, routine DHIS2 data from public facilities documented the coexistence of communicable and non-communicable conditions within everyday service utilization, with NCDs increasing with age while communicable conditions remained prominent among younger adults (Moramarco et al.). This makes the double burden visible in ordinary care, not only in global estimates. In South Africa, the DIMAMO surveillance site shows that epidemilogical transition is socially patterned: smoking, physical inactivity, and low fruit and vegetable intake are concentrated among poorer groups, whereas diabetes and alcohol use are more common among wealthier groups (Ravhuhali et al.). Breast cancer projections add a future-oriented dimension, showing uneven growth in the cancer burden and persistent mortality-to-incidence gaps in areas where access to early diagnosis and treatment is limited (Freihat et al.). Together, these studies show that transition unfolds through local combinations of age, sex, socioeconomic position, disease category, and service capacity. This heterogeneity makes health-system adaptation, rather than disease-specific expansion alone, the central task of such a transition.

The articles on service delivery and policy in this Research Topic demonstrate that this task requires more than adding NCD activities to stretched systems. In Rwanda, pharmacy-based screening illustrates how community pharmacies can become accessible entry points for diabetes, hypertension, and obesity detection, especially for individuals who may not routinely use preventive services; it also shows that screening matters only when linked to follow-up, diagnosis, treatment, and financing (Chiu et al.). In the Central African Republic, structured hypertension care in Bangui suggests that chronic disease management can be feasible in fragile settings; however, difficulties in achieving control among patients with diabetes and chronic kidney disease underscore the need for continuity and comorbidity-sensitive pathways (Doro Altan et al.). Incorporating mental health and substance use care into India's NCD services expands the focus from disease silos to patient-centered care (Balhara et al.). Indonesia's cardiovascular-kidney-metabolic framework similarly points to coordinated clinical and policy responses for conditions that share biological, behavioral, and social pathways (Nasution et al.). Across these examples, connected services depend on clinical redesign and on information systems that make needs, referrals, and outcomes visible.

Health-system adaptation also depends on what systems are able to measure. The indicator-focused study by Agarwal et al. argues that screening and prevention programs need measures that capture not only activity but also timeliness, quality, coverage, and effectiveness, as reflected in the proposed Screening Index and Prevention Index (Agarwal et al.). Sobers et al. extend this logic to NCD registry development in Small Island Developing States, showing that useful data systems require human resources, financing, information infrastructure, data governance, and implementation readiness. Along with the Iraqi Kurdistan experience, in which routine digital data made the double burden visible in everyday services (Moramarco et al.), these contributions show that measurement is integral to public health initiatives. The lessons emerging across this Research Topic are therefore as much organizational as epidemiological, pointing to the importance of institutions that can generate, use, and learn from data.

Several lessons emerge across these diverse settings. First, the double burden is context-specific: it varies by age, sex, socioeconomic position, comorbidity, and service capacity. Second, social inequalities shape both exposure to risk and access to diagnosis and follow-up. Third, primary care remains a key platform for managing the epidemilogical transition, provided it can offer continuity, referrals, medications, and trust. Community platforms, including pharmacies, can extend reach while measurement systems can support learning and accountability. No single model emerges, but the requirements are shared: connected services, implementation capacity, and attention to patients with multiple needs.

Looking ahead, the challenge for LMICs is not to replace infectious disease programs with parallel NCD programs. Evidence from HIV programs in sub-Saharan Africa suggests that disease-specific platforms can also strengthen broader chronic care when adapted for integrated service delivery (5). Similarly, this Research Topic points to resilient, connected, and people-centered systems that can address communicable diseases, chronic conditions, mental health needs, and social vulnerability throughout the life span. These systems will need to be locally adapted, equity-oriented, and informed by routine data. In this next phase of the epidemiological transition, progress will depend less on prioritizing individual diseases than on building systems capable of managing their coexistence.
